# Emergence of *Serratia nevei* co-producing NDM-1 and CTX-M-15 in urban wastewater

**DOI:** 10.1128/spectrum.01378-25

**Published:** 2025-10-22

**Authors:** João Pedro Rueda Furlan, Giovanna Carrasco Bueno, Rubens Renato Sousa-Carmo, Renan Lourenço Oliveira Silva, Mikaela Renata Funada Barbosa, Maria Ines Zanoli Sato, Nilton Lincopan, Sergio Schenkman

**Affiliations:** 1 Paulista School of Medicine, Federal University of São Paulo 28105, São Paulo, Brazil; 2 Antimicrobial Resistance Institute of São Paulo (ARIES), São Paulo, Brazil; 3 Department of Microbiology, Instituto de Ciências Biomédicas, University of São Paulo 525539, São Paulo, Brazil; 4 Department of Environmental Analysis, Environmental Company of São Paulo State (CETESB) 359207, São Paulo, Brazil; 5 Department of Clinical Analysis, Faculty of Pharmacy, University of São Paulo 505153, São Paulo, Brazil; Instituto de Higiene, Montevideo, Uruguay

**Keywords:** *Enterobacteriaceae*, carbapenemase, ESBL, plasmid analysis, wastewater-based surveillance

## LETTER

Carbapenem-resistant *Enterobacterales* (CRE) have become a major public health concern. While clinically relevant CRE species, such as *Klebsiella pneumoniae* and *Escherichia coli*, are the most reported carriers of carbapenemases (1), unusual CRE species are emerging. *Serratia nevei*, a novel species from the *Serratia marcescens* complex (SMC) (2), was first described from cucumber in Germany (3). Recently, *S. nevei* strains, including those producing VIM-1 metallo-β-lactamase or OXA-48 class D β-lactamase, were identified in a Spanish hospital setting as causing human infections or colonizing sinks (2, 4). In the environmental sector, wastewater-based surveillance has been recognized as an affordable and non-invasive method for monitoring emerging pathogens, offering an early alert of human transmission before it is identified through clinical surveillance (5). Herein, we report the emergence of *S. nevei* strain co-producing NDM-1 and CTX-M-15 in urban wastewater in Brazil.

During a local surveillance study conducted in 2024 to monitor the occurrence of WHO critical priority *Enterobacterales* (6) in wastewater samples, a CRE strain (designated M10) was recovered from a sewage treatment plant in São Bernardo do Campo (23°46′39.5″S 46°31′59.8″W), the largest city in the Southeast Zone of São Paulo, which is the most populous and largest city area in South America. Strain M10 was initially identified as *S. marcescens* by Bruker’s matrix-assisted laser desorption/ionization time-of-flight mass spectrometry.

This strain exhibited a multidrug-resistant profile to the novel imipenem-relebactam, ceftazidime-avibactam (MIC >256/4 mg/L), and ceftolozane-tazobactam β-lactam/β-lactamase inhibitor combinations, and meropenem (MIC >64 mg/L), imipenem, ceftriaxone (MIC >64 mg/L), ceftazidime, cefepime, aztreonam, ciprofloxacin, gentamicin, amikacin, minocycline, and chloramphenicol, but remained susceptible to cefiderocol, tetracycline, tigecycline (MIC 2 mg/L), and trimethoprim-sulfamethoxazole, as determined by disk diffusion, broth microdilution, or gradient diffusion strip methods according to the guidelines of CLSI (M100, 34th ed., 2024) and EUCAST (v.14.0, 2024). Metallo-β-lactamase production was confirmed using the meropenem-EDTA disk test.

Strain M10 was sequenced using Illumina HiSeq and Oxford Nanopore Technologies after having its genomic DNA extracted by the PureLink Genomic DNA Mini Kit (Thermo Fisher Scientific, USA). The genome was *de novo* hybrid assembled by Unicycler v.0.5.1 (https://github.com/rrwick/Unicycler). Average nucleotide identity (ANI) and digital DNA–DNA hybridization (dDDH) analyses were used for species-level identification. Multilocus sequence typing was determined by PubMLST for *Serratia* spp. (https://pubmlst.org/organisms/serratia-spp). Single nucleotide polymorphism (SNP) was performed by CSI Phylogeny v.1.4 (https://cge.food.dtu.dk/services/CSIPhylogeny/) using the LMG 31536 genome (BioSample: SAMN40188831) as reference. Antimicrobial resistance genes (ARGs) and plasmid replicons were identified using ResFinder v.4.6.0 (http://genepi.food.dtu.dk/resfinder) and PlasmidFinder v.2.1 (https://cge.food.dtu.dk/services/PlasmidFinder/), respectively. Virulence determinants were identified *in-house* using Geneious Prime 2025.0.2 (Biomatters Ltd., New Zealand) (7).

The ANI and dDDH values between the M10 strain (BioSample SAMN47433515) and all validly published *Serratia* type strains available in April 2025 were computed, confirming the M10 strain as *S. nevei* (ANI 97.34% and dDDH 92.71%). Due to the complex taxonomy of SMC, the misidentification of *S. nevei* as *S. marcescens*, including those lineages producing IMP-4 metallo-β-lactamase (8), has been observed in the NCBI Assembly Database. The strain M10 belonged to sequence type (ST) 732, a clone previously isolated from a human in the United States and identified as harboring the *bla*_KPC-3_ gene (BioSample: SAMN12440941). Notably, in Brazil, there has been only one report of *S. nevei*, with the 9rpt1 carbapenem-susceptible strain being isolated in 2019 from a blackwater river in the Amazon region (9). Based on whole-genome SNP-based analysis, the M10 strain differed from the 9rpt1 strain (GenBank accession number JAMYWQ010000000) by 70,868 SNPs, underscoring distinct genomic lineages linked to different ecological niches.

**Fig 1:**
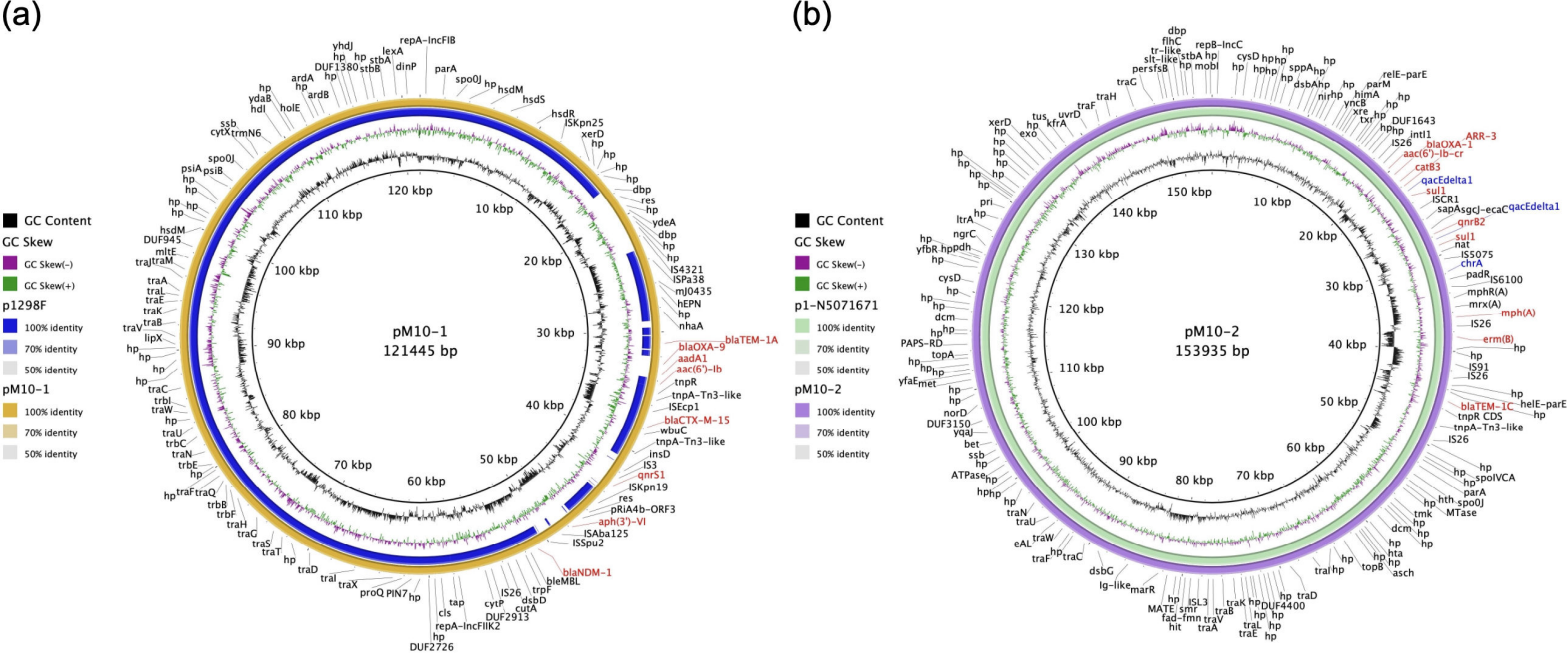
Plasmids IncFIIB/IncFII_K2_ and IncC3 carrying ARGs in *S. nevei* strain M10. (**a**) Comparison between pM10-1 (this study) and p1298F (GenBank accession number MW363916) from *K. pneumoniae* of Poland. (**b**) Comparison between pM10-2 (this study) and p1-N5071671 (GenBank accession number CP165808) from *K. pneumoniae* of Brazil. ARGs and biocide/metal tolerance genes are highlighted in red and blue colors, respectively. Hypothetical protein, hp. Plasmid visualization was performed using BRIG (https://sourceforge.net/projects/brig/).

Strain M10 carried a wide range of acquired antimicrobial resistance (AMR) and biocide/metal tolerance determinants. In this regard, genes *bla*_NDM-1_, *bla*_CTX-M-15_, *bla*_OXA-9_, *bla*_TEM-1A_, *qnrS1*, *aac(6')-Ib*, *aph(3')-VI*, and *aadA* were identified on an IncFIIB/IncFII_K2_ plasmid (pM10-1, 121,445 bp), while *bla*_OXA-1_, *bla*_TEM-1C_, *qnrB2*, *aac(6')-Ib-cr*, *catB3*, *sul1*, *ARR-3*, *mph(A*), *erm(B*), *qacEdelta1* (biocide tolerance), and *chrA* (chromate tolerance) were carried by an IncC3 plasmid (pM10-2, 153,935 bp). Plasmids pM10-1 and pM10-2 shared high nucleotide identity with others isolated from clinical *K. pneumoniae* strains from Poland (10) (Fig. 1a) and Brazil (Fig. 1b), respectively. The *bla*_NDM-1_ and *bla*_CTX-M-15_ genes were associated with Tn*125*-like and IS*Ecp1* elements, respectively, which have been linked to the mobilization of clinically important ARGs worldwide. Interestingly, the 9rpt1 strain harbored only intrinsic ARGs, including *bla*_SRT_, *aac(6′)-Ic*, *tet(41*), hypothesizing that interactions within local microbial communities may play a role in acquiring ARGs, which warrants further investigation.

The transferability and stability of the *bla*_NDM-1_-bearing pM10-1 plasmid were evaluated by conjugation assay and daily serial passages, respectively. Accordingly, transconjugants were not observed, and the pM10-1 plasmid remained stable over 400 generations. Despite this plasmid carrying a putative *oriT* region and the core conjugation determinants (Relaxase, T4SS, and T4CP gene cluster), the absence of annotated auxiliary proteins may explain the lack of transfer observed *in vitro*, and conjugation efficiency may also be influenced by growth phase, temperature, and nutrient-dependent conditions (11). The long-term stability of the pM10-1 plasmid highlights its potential to act as a persistent environmental reservoir of ARGs, posing a risk for the maintenance and indirect dissemination of AMR in microbial communities. Furthermore, important virulence mechanisms of the clinical *S. marcescens* species complex, encoded by genes *shlA*, *shlB*, *phlA*, *lipB, lipC*, *lipD*, *pigP*, *flhC*, and *flhD*, were also detected in the M10 strain, indicating a putative high pathogenic profile (7).

In summary, we report a wastewater-related *S. nevei* strain harboring carbapenemase and extended-spectrum β-lactamase genes as an emergent WHO critical priority bacterial pathogen. Considering that *S. nevei* is emerging as a producer of carbapenemases, including those that degrade novel β-lactams and β-lactam–β-lactamase inhibitor combinations, a need for enhanced surveillance, molecular characterization, and taxonomic resolution of the SMC remains necessary to better understand its contribution to the expanding threat of carbapenem resistance. These findings highlight the potential of wastewater-based epidemiology to detect emerging antimicrobial-resistant pathogens, providing actionable data to guide the design of AMR surveillance programs.

## Data Availability

The genome sequences of *S. nevei* strain M10 are available at GenBank under accession numbers CP186928 (chromosome), CP186929 (plasmid pM10-1), and CP186930 (plasmid pM10-2).
